# Multimechanistic Antifibrotic Effect of Biochanin A in Rats: Implications of Proinflammatory and Profibrogenic Mediators

**DOI:** 10.1371/journal.pone.0069276

**Published:** 2013-07-16

**Authors:** Randa M. Breikaa, Mardi M. Algandaby, Ebtehal El-Demerdash, Ashraf B. Abdel-Naim

**Affiliations:** 1 Department of Pharmacology & Toxicology, Faculty of Pharmacy, Ain Shams University, Cairo, Egypt; 2 Department of Biology (Botany), Faculty of Science, King Abdulaziz University, Jeddah, Saudi Arabia; National Institutes of Health, United States of America

## Abstract

**Objective:**

Biochanin A (BCA) is an isoflavone found in red clover and peanuts. Recently, it drew much attention as a promising anticancer and antioxidant. Due to its diversity in pharmacological actions, we were encouraged to investigate its potential as an antifibrotic, elucidating the different molecular mechanisms involved.

**Design:**

Rats were pretreated with BCA, then injected with carbon tetrachloride (CCl_4_) for 6 weeks. Changes in liver weight and histology were examined and levels of aspartate and alanine aminotransferases, cholesterol, triglycerides, alkaline phosphatase and total bilirubin measured. To assess hepatic efficiency, indocyanine green was injected and its clearance calculated and albumin, total proteins and insulin-like growth factor-1 expression were measured. Cytochrome P4502E1 activity, cytochrome P4501A1 expression, in addition to sulfotransferase1A1 expression were determined to deduce the effect of BCA on hepatic metabolism. As oxidative stress markers, lipid peroxides levels, reduced glutathione, superoxide dismutase and catalase activities, as well as the total antioxidant capacity, were assessed. Nitric oxide, inducible nitric oxide synthase and cyclooxygenase-2 were used as indicators of the inflammatory response. Signaling pathways involving tumor necrosis factor-alpha, nuclear factor-kappa B, transforming growth factor-beta1, matrix metalloproteinase-9 and alpha-smooth muscle actin were investigated accordingly. Extent of fibrosis was examined by Masson’s stain and measuring hydroxyproline levels.

**Results:**

BCA pretreatment significantly protected against the chronic damage of CCl_4_. Liver injury, oxidative stress, inflammation and fibrosis markers decreased, while hepatic efficiency improved.

**Conclusion:**

Our findings suggested that BCA administration protects against fibrotic complications, a property that can be contributed to the multimechanistic approach of the drug.

## Introduction

Chronic liver diseases represent a major public health problem worldwide. Their prognosis greatly depends on the extent of progression leading to serious complications such as fibrosis [1]. Hepatic fibrosis is a dynamic process characterized by imbalance in production and degradation of extracellular matrix (ECM) components in the liver. The fibrotic process involves various cells and factors that lead to disruption of intercellular contacts, extracellular matrix composition and liver ability to regenerate [2]. Because of significant progress in uncovering its pathogenetic mechanisms, combined with a growing realization that effective antifibrotic therapies may soon alter the history of chronic liver diseases, many candidates are now being investigated as potential therapies for fibrosis [3]. However, a major obstacle in the development of new antifibrotic drugs is the long-term use of the drugs which dictates absence of toxicities associated with the medications. This property gives natural compounds a profound advantage [4].

Flavonoids are phenolic compounds that represent vital constituents of the human diet. With their hydroxyl groups, they possess strong antioxidative activity, making them perfect candidates to protect against various diseases [5]. Their importance is further substantiated by the fact that they do not act as conventional hydrogen-donating antioxidants but may exert modulatory actions in cells through actions at various signaling pathways [6]. A clear understanding of their mechanisms of action is key to the evaluation of these potent biomolecules in treatment and prophylaxis.

BCA is an O-methylated isoflavone. It is a natural compound found in red clover, soy, alfalfa sprouts, peanuts and chickpea. A broad spectrum of biological benefits has been reported, including anti-inflammatory, antioxidative and antineoplastic effects [7].

Based on the ever-increasing list of the beneficial effects of flavonoids, and the present day need for an effective, economical and simple strategy for reversing liver injury, we planned the present study. The potential of BCA to act as antifibrotic was assessed by tackling different molecular mechanisms including effects on tumor necrosis factor-alpha (TNF-α), nuclear factor-kappa B (NF-κB) and transforming growth factor-beta1 (TGF-β1) which are attractive targets for therapeutic interventions as they represent cross-talks between cells [8].

## Materials and Methods

### Chemicals

BCA, CCl_4_, dimethylsulfoxide (DMSO), p-nitrophenol, p-nitrocatechol, Ellman’s reagent, chloramine-T, p-dimethylaminobenzaldehyde, indocyanine green (ICG) and thiobarbituric acid (TBA) were purchased from Sigma Chemical Co., St Louis, MO, USA. Reduced nicotinamide adenine dinucleotide phosphate (NADPH) was supplied by Sorachim Chemicals, Lausanne, Switzerland. All other chemicals used were of highest grade commercially available.

### Animals and Experimental Protocol

The study was conducted on 200–250 g male Wistar rats, supplied by the Animal Breeding Laboratory, Helwan, Egypt. All conditions were in accordance with the Animal Research: Reporting of In Vivo Experiments (ARRIVE) guidelines, developed by the National Centre for the Replacement, Refinement and Reduction of Animals in Research (NC3Rs) and approved by the ethical committee of Ain Shams University, Egypt.

The animals were randomly divided into four groups of eight rats each. Group A served as control, taking DMSO and corn oil on alternative days and group B as the hepatotoxic model, taking intraperitoneal (IP) CCl_4_ (1 ml/kg, 1∶1 mixture with corn oil) twice weekly. Group C was given both CCl_4_ and BCA (50 mg/kg, IP dissolved in DMSO) three times per week on alternating days with CCl_4_. The dose was selected on the basis of preliminary studies, including determination of the median lethal dose (LD_50_) and the least effective hepatoprotective dose [9]. Group D was given BCA alone. All groups were injected over a period of six weeks, then blood samples were collected from retro-orbital plexus and serum was separated by centrifugation at 1000 g for 10 min and was used for the assessment of liver functions. Rats were sacrificed by decapitation 24 h after the last injection and livers were removed and weighed, then divided into three parts: one for homogenization in saline, one put in formalin for immunohistochemical and histopathological analysis, and the last part kept as such and put together with the 20% homogenate in −80°C until needed.

### Hepatic Integrity Markers and Liver Index

Serum aspartate aminotransferase (AST), alanine aminotransferase (ALT), total cholesterol (TC), triglycerides (TG), alkaline phosphatase (ALP) and total bilirubin were estimated colorimetrically using available commercial kits (Spectrum diagnostics, Cairo, Egypt). Liver index was calculated according to the formula: (liver weight/body weight)×100.

### Assessment of Hepatic Efficiency

#### Blood flow

At the end of the six weeks, six animals per group were injected with a single dose of 3 mg/kg ICG, IP, dissolved in saline. Blood samples were collected after 10 min, 30 min, 1 h and 2 h. The plasma was separated and its optical density measured spectrophotometrically at 805 nm. The elimination rate constant (β), clearance (Cl), half-life (t_1/2_), area under the curve (AUC) and volume of distribution (Vd) were calculated. Serial dilutions of ICG (from 2.5–50 µg/ml) were prepared to construct a standard calibration curve.

#### Synthetic capacity

Serum albumin and total proteins (TP) were assessed colorimetrically using commercial kits (Spectrum diagnostics, Cairo, Egypt), while insulin-like growth factor-1 (IGF-1) was assessed in homogenate using enzyme-linked immunosorbent assay (ELISA) kit from R&D Systems, USA.

#### Metabolic capacity

Cytochrome P450 2E1 (CYP2E1) activity was assessed in homogenate colorimetrically by monitoring the formation of p-nitrocatechol from p-nitrophenol by CYP2E1. The enzymatic product, p-nitrocatechol, was assayed at 535 nm after acidification of the reaction mixture with trichloroacetic acid (TCA) followed by neutralization using 2 N NaOH [10]. Assessment of cytochrome P450 1A1 (CYP1A1) expression in homogenate was done using ELISA kits from Cusabio Biotech., China. Sulfotransferase 1A1 (SULT1A1) expression was estimated by real time-polymerase chain reaction (RT-PCR). RNA was extracted from liver according to manufacturer’s protocol (Qiagen, Hilden, Germany), then reverse transcribed and RT-PCR was performed using SYBR Green. Beta-Actin (β-Actin) served as internal control. Primers used were: *ratACTBF320∶5′-AGGCCCCTCTGAACCC TAAG-3′, ratACTB-R435∶5′-AGAGGCATACAGGGACAACACA-3′; rSULT1A1-F530∶5′-AGCTGAGACACACTCACCCTGTT-3′, rSULT1A1-R651∶5′-ATCCACAGTCTCCTCGGGTAGA-3′* [11].

### Assessment of Oxidative Stress

Lipid peroxidation was determined in homogenate by estimating level of thiobarbituric acid reactive substances (TBARS) measured as malondialdehyde (MDA), according to the method of Mihara [12]. To determine reduced glutathione (GSH), 0.5 ml homogenate was added to 0.5 ml of 10% TCA. The tubes were shaken intermittently for 15 min, followed by centrifugation at 1000 g for 10 min. An aliquot of the resulting supernatant (0.2 ml) was added to a tube containing 1.7 ml phosphate buffer and 0.1 ml Ellman’s reagent then the absorbance was read at 412 nm [13]. Superoxide dismutase (SOD) and catalase (CAT) activities were determined in homogenate using commercial kits (Biodiagnostics, Cairo, Egypt). In addition, the total antioxidant capacity (TAC) was measured by providing a specific amount of exogenous H_2_O_2_ which is then reduced by antioxidants in the sample. The residual H_2_O_2_ was determined colorimetrically.

### Assessment of Inflammatory Response

TNF-α expression was assessed using ELISA kit from RayBiotech., Inc., USA. Total nitric oxide (NO) content was estimated in homogenate spectrophotometrically by Miranda’s method [14] and results were expressed as µmol/g wet tissue. Inducible nitric oxide synthase (iNOS) and cyclooxygenase-2 (COX-2) expressions were examined immunohistochemically. Liver sections of 4 µm were cut, then after preparing the slides, one of the following ready-to-use primary antibodies was applied: rabbit polyclonal antibody to rat iNOS (Thermoscientific, iNOS Cat#RB-9242-R7) or rabbit polyclonal anti-rat COX-2 (Thermoscientific, COX-2 Cat#RB-9072-R7). Examination was done with a light microscope.

### Assessment of NF-κB

NF-κB expression was examined immunohistochemically using rabbit polyclonal IgG to rat NF-κB p65 (SantaCruz Biotech, Cat No. sc-372).

### Assessment of Fibrosis Markers

TGF-β1 was assessed using ELISA kit from R&D Systems, USA. Matrix metalloproteinase-9 (MMP-9) and alpha-smooth muscle actin (α-SMA) were examined immunohistochemically using goat polyclonal IgG to rat MMP-9 (SantaCruz Biotech, MMP-9 Cat No. sc-6840) and mouse monoclonal to rat α-SMA (SantaCruz Biotech, Cat No. sc-53142). To examine the extent of fibrosis, hydroxyproline was estimated in the liver using the simplified method of Reddy [15]. In addition, Masson’s trichrome stain was used for collagen fiber detection.

### Histopathologic Examination

Liver sections were stained with hematoxylin and eosin (H&E) and examined by light microscope.

### Statistical Analysis

Data is presented as mean ± standard deviation (SD). Multiple comparisons were performed using one-way ANOVA followed by Tukey–Kramer as a post-hoc test. Differences between groups were considered significant at p<0.05. All statistical analyses were performed using Instat software package (version 3.06). Graphs were sketched using GraphPad Prism software (version 5).

## Results

### Hepatic Integrity and Liver Index

AST and ALT increased after chronic CCl_4_ challenge by 1.8 and 1.5, respectively, compared to control group. TC and TG doubled, while levels of ALP and total bilirubin increased by 1.7 and 1.5 folds (**Table 1**). Pretreatment with BCA normalized these levels, while BCA alone showed levels below the control group. The liver index increased in CCl_4_ group by 1.5 and decreased significantly by BCA pretreatment (**Table 1**).

**Table 1:** Effect of BCA on hepatic integrity markers and liver index.

Group	AST (U/L)	ALT (U/L)	TC (mg/dl)	TG (mg/dl)	ALP (U/l)	Total bilirubin (mg/dl)	Liver index (%)
Control	54.3±9.29***	52.4±3.73***	59.5±7.58***	52.7±10.13***	106.7±5.22***	2.6±0.21**	2.9±0.1***
CCl_4_	80.4±11.93	95±26.47	108.2±22.78	112.6±38.55	181.1±42.8	3.8±0.95	4.3±0.41
BCA+CCl_4_	48±10.26***	69.2±7.27**	69.4±10.95***	73±17.48**	107.7±11.88***	2.6±0.41**	3.8±0.19**
BCA	42.1±4.55***	50±8.07***	59.7±11.31***	43.7±14.5***	101.5±9.31***	2.5±0.58***	2.9±0.29***

n = 8.

* p<0.05 compared to CCl_4_ group.

** p<0.01 compared to CCl_4_ group.

*** p<0.001 compared to CCl_4_ group.

### Hepatic Efficiency

#### Blood flow

ICG elimination rate decreased to the half by CCl_4_ challenge, while the clearance decreased by 23%, compared to control group. Its t_1/2_, AUC and Vd significantly increased by about 4 folds, 1.4 folds and 3.2 folds, respectively (**Table 2**). BCA pretreatment, however, increased the elimination and the clearance by 1.5 folds and 1.2 folds, as compared to CCl_4_ group. T_1/2_, AUC and Vd decreased with BCA by 3, 1.3 and 2.7 folds in comparison to CCl_4_ group.

**Table 2:** Effect of BCA on ICG pharmacokinetics.

Group	β (hr^−1^)	t_1/2_ (hr)	AUC (hr.µg/ml)	Cl (ml/kg/hr)	Vd (ml/kg)
Control	39±5.4***	0.026±0.008***	209.2±24.75**	15.3±0.97***	0.6±0.21***
CCl_4_	18.7±2.34	0.101±0.023	291.1±41.6	11.8±0.39	1.9±0.62
BCA+CCl_4_	27.5±6.1	0.032±0.007***	223.3±36.82*	14.6±2**	0.7±0.19***
BCA	32.4±9.93**	0.029±0.008***	227±28.93*	14.2±1.43*	0.5±0.13***

β (elimination rate constant), Cl (clearance), t_1/**2**_ (elimination half-life) and AUC (area under the curve).

n = 6.

* p<0.05 compared to CCl_4_ group.

** p<0.01 compared to CCl_4_ group.

*** p<0.001 compared to CCl_4_ group.

#### Synthetic capacity

Albumin, TP and IGF-1 were depleted by chronic CCl_4_ intoxication, where TP reached half and IGF-1 1/16 of the control levels. BCA pretreatment significantly prevented their depletion (**Table 3**).

**Table 3:** Effect of BCA on hepatic synthetic capacity.

Group	Albumin (g/dl)	TP (g/dl)	IGF-1 (pg/mg protein)
Control	3.1±0.52	6.6±1.06***	2674.5±319.87***
CCl_4_	2.4±0.43	3.4±0.99	161.4±53.51
BCA+CCl_4_	3.3±0.91*	5.4±0.81***	1053.8±144.63***
BCA	3.4±0.31*	5.6±0.81***	2583.9±331.63***

n = 8.

* p<0.05 compared to CCl_4_ group.

** p<0.01 compared to CCl_4_ group.

*** p<0.001 compared to CCl_4_ group.

#### Metabolic capacity

**Figure 1:**
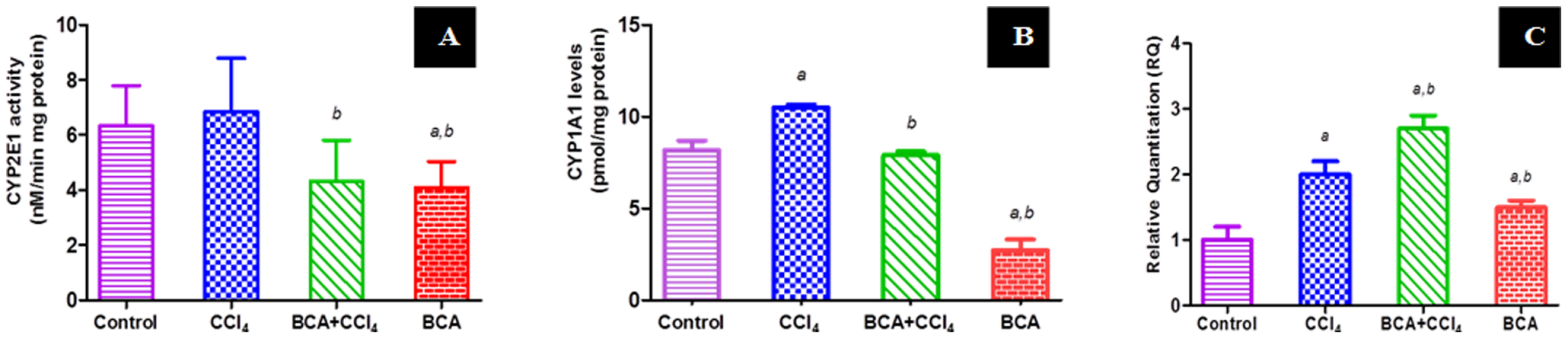
Effect of BCA on hepatic metabolic capacity. (**A**) CYP2E1 activity levels; (**B**) CYP1A1 expression levels; (**C**) SULT1A1 relative quantitation (RQ) versus group mean plot. *a*: significantly different from control gp. *b*: significantly different from CCl_4_ gp.

The activity of CYP2E1 increased in CCl_4_ group by 10%, as compared to control (**Fig. 1**). The pretreatment with BCA significantly reduced the activity even below levels of control.

In addition, the expression of CYP1A1 was significantly higher after chronic CCl_4_ challenge, while it approached normal levels by BCA pretreatment (**Fig. 1**). The group treated with BCA alone showed levels that reach one third of control group. RT-PCR results showed increased expression of SULT1A1 in all groups compared to control (**Fig. 1**). However, increases in CCl_4_ and pretreated group were higher than BCA alone suggesting that not only BCA increased the expression, but also the CCl_4_ challenge did.

### Oxidative Stress Markers

CCl_4_ increased MDA level by 62%, while GSH was severely depleted, reaching about 11% of the control level. The TAC decreased by 41%, as compared to control group (**Table 4**). Animals pretreated with BCA showed near normal MDA and TAC levels. The dramatic depletion of GSH was reversed and even increased by 19% above control, while BCA alone increased GSH level by 31%. Furthermore, SOD and CAT activities were suppressed by CCl_4_ and returned to normal levels by BCA (**Table 4**).

**Table 4:** Effect of BCA on oxidative stress markers.

Group	MDA (nmol/g)	GSH (µmol/g)	TAC (µmol/g)	SOD (U/mg)	CAT (U/g)
Control	11±1.32***	6±2.1***	0.0032±0.0005***	55.7±11.71***	898.9±56.55***
CCl_4_	17.7±2.81	0.7±0.23	0.0019±0.0006	21±6.31	640±73.72
BCA+CCl_4_	11.6±0.96***	7.2±2***	0.0033±0.0002***	51.5±7.8***	899.1±85.63***
BCA	11.5±0.74***	7.9±1.97***	0.0035±0.0002***	51.4±6.3***	896.7±40.95***

n = 8.

* p<0.05 compared to CCl_4_ group.

** p<0.01 compared to CCl_4_ group.

*** p<0.001 compared to CCl_4_ group.

### Inflammation Markers

**Figure 2:**
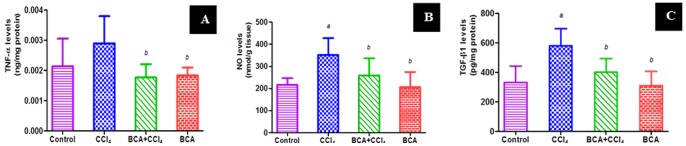
Effect of BCA on TNF-α, NO and TGF- β1 levels. (**A**) TNF-α expression levels; (**B**) NO levels; **(C)** TGF- β1 expression levels. *a*: significantly different from control gp. *b*: significantly different from CCl_4_ gp.

**Figure 3:**
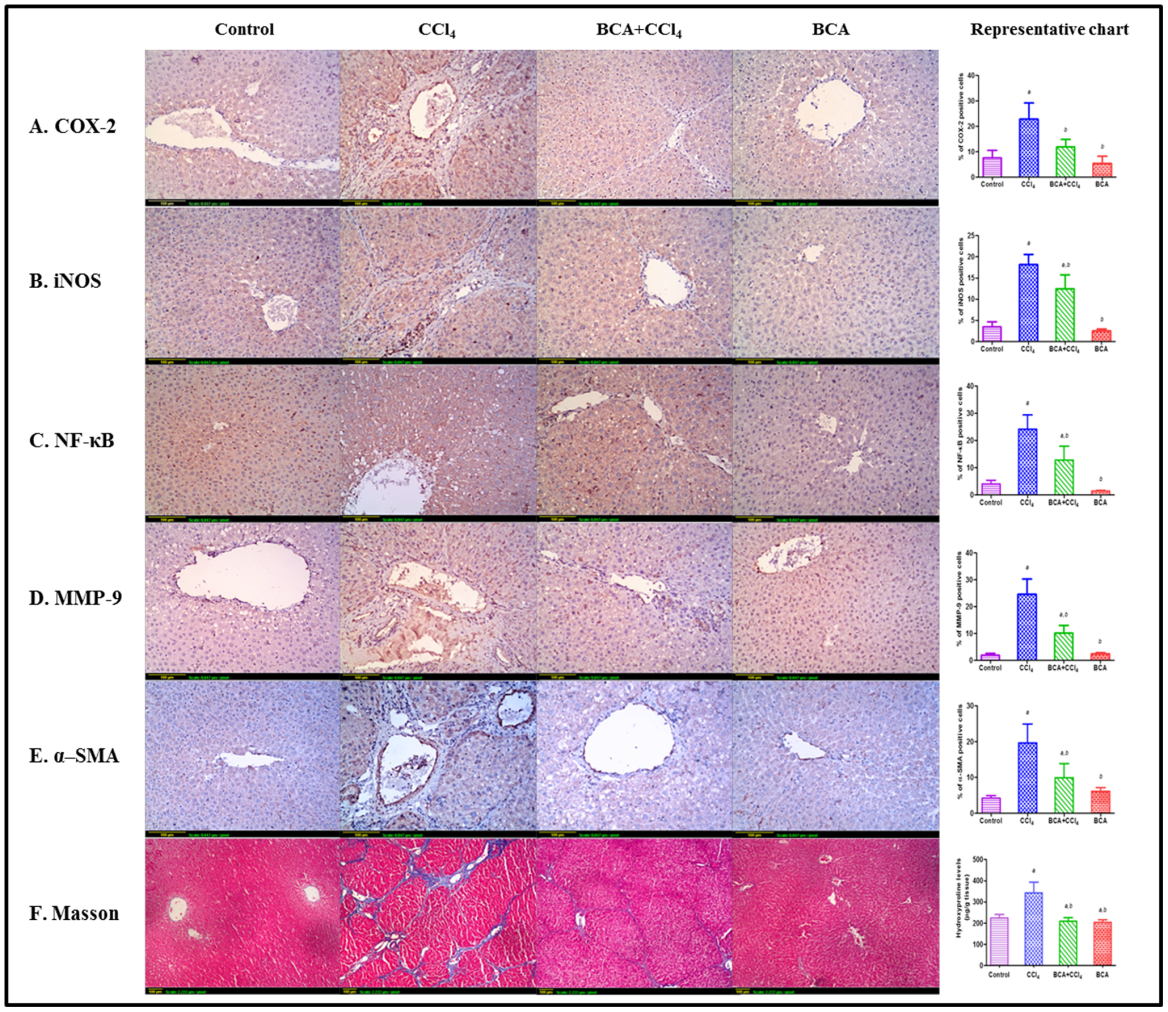
Representative images of liver sections of different experiments. (**A–E**): Immunohistochemical detection of COX-2, iNOS, NF-κB, MMP-9 and α-SMA (x100). Control gp: minimal expression; CCl_4_ gp: extensive expression; BCA+CCl_4_ gp: less than CCl_4_ gp; BCA gp: minimal expression. Image analysis was performed by examining 6 fields/slide. (**F**): Masson trichrome stain (x40). Control gp: shows absence of collagen fibers (stained blue) between hepatic lobules; CCl_4_ gp: extensive fibers deposition with pseudolobules formation (bridging fibrosis); BCA+CCl_4_ gp: less fibers than CCl_4_ gp; BCA gp: absence of fibers. The bar chart represents levels of hydroxyproline expressed as µg/gm of wet tissue measured by Reddy’s method. *a*: significantly different from control gp. *b*: significantly different from CCl_4_ gp.

TNF-α and total NO in CCl_4_ group reached 138% and 163% of control group, respectively. BCA restored their levels (**Fig. 2 A & B**). Immunohistochemical staining showed extensive expression of COX-2 and iNOS in CCl_4_ group, while the pretreated group showed marked reduction (**Fig. 3 A & B**). The control and BCA model group showed minimal expression.

### Effect on NF-κB

Control rats showed minimal immunostaining (**Fig. 3 C**), while CCl_4_ group showed significant expression. Pretreatment with BCA significantly inhibited its expression. BCA alone showed expression levels below control group.

### Fibrosis Markers

Fibrosis was first evaluated by measuring TGF-β1 expression which increased by 1.8 folds in CCl_4_ group, while levels decreased by 30% by BCA pretreatment, compared to the CCl_4_ group (**Fig. 2 C**). Immunohistochemistry revealed high MMP-9 (**Fig. 3 D**) and α-SMA (**Fig. 3 E**) in CCl_4_ group. Furthermore, high hydroxyproline levels were detected with dense blue color in Masson’s slides around and inbetween portal tracts and central veins, indicating pseudolobules formation (**Fig. 3 F**). Significantly lower levels were found in the other groups.

### Histopathology

**Figure 4:**
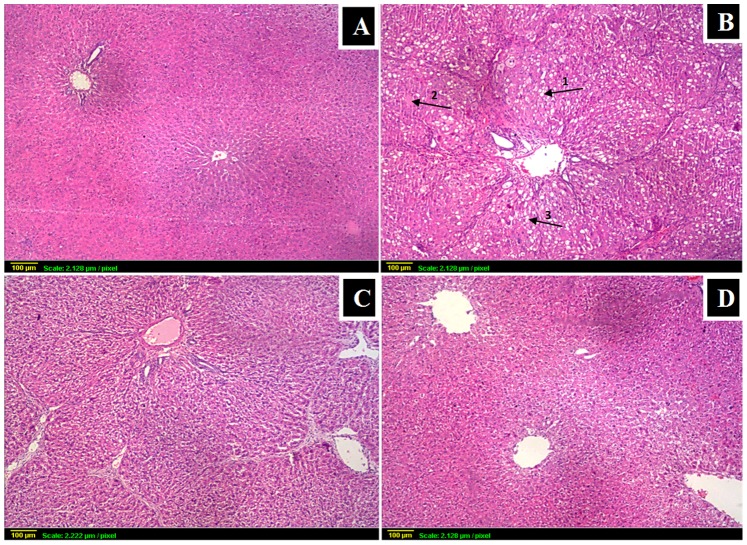
Histopathological findings followed by grading of liver damage. (x40) (**A**) control: normal histological structure of portal area and surrounding hepatocytes; (**B**) CCl_4_: loss of architecture with severe ballooning degeneration (arrow1), necrosis (arrow2) and fatty changes (arrow3) accompanied with fibrosis; (**C**) BCA+CCl_4_: much less damage than in CCl_4_ gp (**D**) BCA alone: similar to control gp.

CCl_4_ caused marked necrosis with fatty deposits and ballooning degeneration, associated with extensive fibrosis and inflammatory cells infiltration (**Fig. 4**). BCA significantly ameliorated these changes. Sections from control and BCA model group showed normal hepatic architecture with polyhedral hepatocytes and prominent nuclei. The histopathological findings were scored by a histopathologist.

## Discussion

Hepatic fibrosis is the consequence of wound-healing response of the liver to repeated injury resulting in accumulation of tough, fibrous scar tissue [16]. It develops with different spatial patterns according to causes of the parenchymal damage. Key events are the propagation of oxidative and inflammatory responses with subsequent transformation of quiescent hepatic stellate cells (HSCs) into myofibroblast-like cells [17].

Flavonoids are phenolic compounds that represent vital constituents of the human diet. With their hydroxyl groups, they possess strong antioxidative activity, making them perfect candidates to protect against various diseases [5]. Their importance is further substantiated by the fact that they do not act as conventional hydrogen-donating antioxidants but may exert modulatory actions in cells through actions at various signaling pathways [6]. A clear understanding of their mechanisms of action is key to the evaluation of these potent biomolecules in treatment and prophylaxis. BCA is a natural isoflavone with multiple pharmacological activities, including antihyperglycemic, anti-inflammatory and antioxidative effects [18], [19]. A study by Badria et al has shown that BCA inhibits HSC proliferation, thus pointing to a potential antifibrotic activity [20].

Events of hepatic fibrosis were simulated in this study using a CCl_4_-induced fibrosis model. First, to ensure development of fibrosis by CCl_4_ and its decrease by BCA, collagen deposition was determined both biochemically and histologically by visualizing the blue color of collagen fibers using Masson’s stain. Both confirmed fibrotic lesions after CCl_4_ challenge and minimal deposition with BCA. In addition, the fact that BCA was able to decrease α-SMA, a marker of activated HSCs, confirms its antifibrotic effect [21].

The mechanisms underlying this antifibrotic activity were then examined thoroughly. High AST, ALT, TC and TG levels confirmed CCl_4_-induced hepatocellular damage. High ALP and total bilirubin indicated dysfunction of hepatocytes and increased biliary pressure. The reversal of these alterations by BCA is a clear indication of the improvement of the functional status of hepatocytes with preservation of cellular architecture [22].

In an attempt to investigate possible changes in liver efficiency, hepatic blood flow, synthetic and metabolic capacities were examined. Effect on blood flow was investigated by assessing changes in ICG pharmacokinetics which is exclusively eliminated by the liver without metabolic modifications or enterohepatic recirculation. Furthermore, its presence in the blood can be accurately assessed in presence of hyperbilirubinemia [23]. Thus, the rate limiting step for ICG elimination is the hepatic blood flow [24]. CCl_4_ clearly decreased the elimination of ICG, reflecting compromised blood flow which can be attributed to the necrotic effect of CCl_4_ causing irreversible damage of microvascular circulation, in addition to excessive deposition of collagen fibers in extracellular spaces, causing hindered blood infusion [25]. BCA successively improved the blood flow. The hepatic synthetic capacity was evaluated by measuring albumin, TP and IGF-1, all of which are synthesized in the liver. Chronic CCl_4_ challenge caused their severe depletion, while BCA restored their levels. Owing to its unique vascular and metabolic features, the liver is exposed to many xenobiotics, which are either activated or detoxified by drug-metabolizing enzymes [26]. CYP2E1 is one principal member of the phase I metabolizing enzymes, the cytochrome P450 (CYP450) family, responsible for bioactivation of many xenobiotics, including CCl_4_ [27]. BCA was found to affect the activity of CYP2E1 and even inhibit it to levels lower than those of the control group, thus, allowing BCA to suppress the bioactivation of various xenobiotics. In addition to CYP2E1, CYP1A1 is another important P450 enzyme causing propagation of liver injury, with marked role in oxidative stress by excessive generation of reactive oxygen species (ROS) [28], [29]. SULT1A1 was chosen as a phase II enzyme helping the liver to get rid of xenobiotics [30]. Our study revealed increase of CYP1A1 expression by chronic CCl_4_ injection associated with decrease by BCA, while SULT1A1 increased both by BCA and CCl_4_, suggesting that sulfotransferases can act as defense against a xenobiotic challenge as CCl_4_.

Oxidative stress produced by free radicals is the main and primary step in CCl_4_ toxicity contributing to both onset and progression of fibrosis [31]. This was evidenced by enhanced lipid peroxidation, associated with low levels of GSH which represents the non-enzymatic part of the antioxidant defense of the body [32]. The enzymatic part was represented in our study with SOD and CAT. The activities of both enzymes were reduced by CCl_4_. Since it became clear that the cooperation among different antioxidants provides greater protection against attack by ROS and free radicals than any single compound alone, the overall antioxidant capacity may provide more accurate information compared to that obtained by the measurement of individual components, as it considers the cumulative effect of all antioxidants present in the liver [33]. As expected, CCl_4_ markedly decreased the TAC. Flavonoids are potent molecules that donate a hydrogen atom from an aromatic hydroxyl group to a free radical, yielding a stable phenolic radical. Furthermore, their amphiphilicity enhances their ability to trap chain-initiating radicals at the interface of the membranes, preventing progression of the radical chain reaction [34]. BCA indeed showed superior antioxidant activity evident by TAC levels above control group.

Another pathway involved in fibrosis is the inflammatory process that is initiated by activation of Kupffer cells, which further release a number of proinflammatory mediators like TNF-α [35]. The heightened inflammatory response following CCl_4_ injection was evident by increased levels of TNF-α, which was significantly attenuated by BCA. NO plays crucial roles in inflammation and liver injury [36]. It is produced in large quantities by Kupffer cells, endothelial cells and the hepatocytes themselves in response to tissue damage and inflammation induced by various xenobiotics including CCl_4_. In addition, its role in oxidative stress cannot be neglected, since high levels of NO have been associated with oxidative injury via lipid peroxide.

production and antioxidant consumption [37]. Our findings confirmed elevated NO in the CCl_4_ group, while BCA caused decreased levels. Two enzymes responsible for initiation and propagation of the inflammatory cascade are iNOS [38] and COX-2 which accounts for the increased production of PGs in response to proinflammatory stimuli [39]. Inhibition of iNOS and COX-2 expressions were found to be two mechanisms by which BCA can act as an anti-inflammatory, thereby protecting the liver. Furthermore, sustained hepatic inflammation provoked by long-term intoxication with CCl_4_ is believed to be through NF-kB pathway [40], [41]. NF-κB is a pleiotropic protein with pivotal role in controlling cell signaling under certain physiological and pathological conditions [42]. In this context, activation of many kinases involved in NF-κB pathway is shown to be dependent on oxidative stress [43], where ROS have been shown to cause prolonged NF-κB DNA binding activity [44], [45]. Further, Kupffer cells display powerful NF-κB activation in response to liver injury by CCl_4_, resulting in production and secretion of proinflammatory cytokines and profibrogenic mediators such as TGF-β1 [35]. TGF-β1 is a multifunctional growth factor stimulating synthesis and deposition of ECM components, in addition to favoring transition of hepatocytes to myofibroblast-like cells [46]. Thus, in our study, both NF-κB and TGF-β1 were found upregulated in fibrotic lesions, while BCA decreased their expression.

Quantitative and qualitative changes in matrix degrading activity play an important role in ECM remodeling and accumulation of ECM components accompanying liver fibrosis [47].

A large family of MMPs has been characterized that is responsible for degrading collagens [48]. However, high levels of MMPs cause excessive degradation of normal subendothelial ECM hastening its replacement by fibrous collagen, which further activates stellate cells in a positive feedback loop [49]. Furthermore, MMP-induced degradation of the ECM releases previously sequestered growth factors that can induce a variety of biological responses including inflammation [50]. One member of this family is MMP-9 which is considered a hallmark of fibrosis [51] and whose expression increases by TNF-α and TGF-β1 during the onset of liver fibrogenesis [52]. MMP-9 increased by CCl_4_, which could be one of the reasons for aggravating its hepatotoxicity.

In light of all the previous findings, the present study provides evidence for the hepatoprotective and antifibrotic effects of BCA. The mechanisms underlying these promising effects involve attenuating oxidative stress, as well as decreasing the expression of NF-κB and the subsequent inflammatory cascade and the production of profibrogenic factors. The net effect is, thus, preservation of the hepatocellular integrity and the hepatic efficiency.

## References

[1] Starkel P, Leclercq IA (2011) Animal models for the study of hepatic fibrosis. Best Pract Res Clin Gastroenterol 25: 319–333. 21497748 10.1016/j.bpg.2011.02.004

[2] Poli G (2000) Pathogenesis of liver fibrosis: role of oxidative stress. Mol Aspects Med 21: 49–98. 10978499 10.1016/s0098-2997(00)00004-2

[3] Dhiman A, Nanda A, Ahmad S (2012) A recent update in research on the antihepatotoxic potential of medicinal plants. Chin J Integr Med 10: 117–127. 10.3736/jcim20120201 22313878

[4] Jordan SA, Cunningham DG, Marles RJ (2010) Assessment of herbal medicinal products: Challenges, and opportunities to increase the knowledge base for safety assessment. Toxicol Appl Pharmacol 243: 198–216. 20018204 10.1016/j.taap.2009.12.005

[5] Williams RJ, Spencer JPE, Rice-Evans C (2004) Flavonoids: antioxidants or signalling molecules? Free Radic Biol Med 36: 838–849. 15019969 10.1016/j.freeradbiomed.2004.01.001

[6] Middleton E Jr, Kandaswami C, Theoharides TC (2000) The effects of plant flavonoids on mammalian cells: implications for inflammation, heart disease, and cancer. Pharmacol Rev 52: 673–751. 11121513

[7] Mishra P, Kale RK, Kar A (2008) Chemoprevention of mammary tumorigenesis and chemomodulation of the antioxidative enzymes and peroxidative damage in prepubertal Sprague Dawley rats by Biochanin A. Mol Cell Biochem. 312: 1–9. 10.1007/s11010-008-9714-8 18273562

[8] Salas AL, Ocampo G, Farina GG, Reyes-Esparza J, Rodriguez-Fragoso L (2007) Genistein decreases liver fibrosis and cholestasis induced by prolonged biliary obstruction in the rat. Ann Hepatol 6: 41–47. 17297428

[9] Breikaa RM, Algandaby MM, El-Demerdash E, Abdel-Naim AB (2013) Biochanin A Protects against Acute Carbon Tetrachloride-Induced Hepatotoxicity in Rats. Biosci Biotechnol Biochem 77: 909–916. 23649249 10.1271/bbb.120675

[10] Chang TK, Crespi CL, Waxman DJ (2006) Spectrophotometric analysis of human CYP2E1-catalyzed p-nitrophenol hydroxylation. Methods Mol Biol 320: 127–131. 16719383 10.1385/1-59259-998-2:127

[11] Chen Y, Huang C, Zhou T, Zhang S, Chen G (2010) Biochanin A induction of sulfotransferases in rats. J Biochem Mol Toxicol 24: 102–114. 20391625 10.1002/jbt.20318

[12] Mihara M, Uchiyama M (1978) Determination of malonaldehyde precursor in tissues by thiobarbituric acid test. Anal Biochem 86: 271–278. 655387 10.1016/0003-2697(78)90342-1

[13] Ellman GL (1959) Tissue sulfhydryl groups. Arch Biochem Biophys 82: 70–77. 13650640 10.1016/0003-9861(59)90090-6

[14] Miranda KM, Espey MG, Wink DA (2001) A Rapid, Simple Spectrophotometric Method for Simultaneous Detection of Nitrate and Nitrite. Nitric Oxide 5: 62–71. 11178938 10.1006/niox.2000.0319

[15] Reddy GK, Enwemeka CS (1996) A simplified method for the analysis of hydroxyproline in biological tissues. Clin Biochem 29: 225–229. 8740508 10.1016/0009-9120(96)00003-6

[16] Friedman SL (2000) Molecular regulation of hepatic fibrosis, an integrated cellular response to tissue injury. J Biol Chem 275: 2247–2250. 10644669 10.1074/jbc.275.4.2247

[17] Parola M, Marra F, Pinzani M (2008) Myofibroblast - like cells and liver fibrogenesis: Emerging concepts in a rapidly moving scenario. Mol Aspects Med 29: 58–66. 18022682 10.1016/j.mam.2007.09.002

[18] Harini R, Ezhumalai M, Pugalendi KV (2011) Antihyperglycemic effect of biochanin A, a soy isoflavone, on streptozotocin-diabetic rats. Eur J Pharmacol 676: 89–94. 22178203 10.1016/j.ejphar.2011.11.051

[19] Kole L, Giri B, Manna SK, Pal B, Ghosh S (2010) Biochanin-A, an isoflavon, showed anti-proliferative and anti-inflammatory activities through the inhibition of iNOS expression, p38-MAPK and ATF-2 phosphorylation and blocking NFkappaB nuclear translocation. Eur J Pharmacol 653: 8–15. 21147093 10.1016/j.ejphar.2010.11.026

[20] Badria FA, Dawidar AA, Houssen WE, Shier WT (2005) In vitro study of flavonoids, fatty acids, and steroids on proliferation of rat hepatic stellate cells. Z Naturforsch C 60: 139–142. 15787259 10.1515/znc-2005-1-225

[21] Teraoka R, Shimada T, Aburada M (2012) The molecular mechanisms of the hepatoprotective effect of gomisin A against oxidative stress and inflammatory response in rats with carbon tetrachloride-induced acute liver injury. Biol Pharm Bull 35: 171–177. 22293346 10.1248/bpb.35.171

[22] Tatiya AU, Surana SJ, Sutar MP, Gamit NH (2012) Hepatoprotective effect of poly herbal formulation against various hepatotoxic agents in rats. Pharmacognosy Res 4: 50–56. 22224062 10.4103/0974-8490.91040 PMC3250040

[23] Hsieh CB, Chen CJ, Chen TW, Yu JC, Shen KL, et al (2004) Accuracy of indocyanine green pulse spectrophotometry clearance test for liver function prediction in transplanted patients. World J Gastroenterol 10: 2394–2396. 15285026 10.3748/wjg.v10.i16.2394 PMC4576295

[24] Tralhao JG, Hoti E, Oliveiros B, Botelho MF, Sousa FC (2012) Study of perioperative liver function by dynamic monitoring of ICG-clearance. Hepatogastroenterology 59: 1179–1183. 22580671 10.5754/hge09726

[25] Suzuki M, Ikeda H, Takahashi H, Okuse N, Kobayashi Y, et al (2003) 545 Hepatic blood flow measurement with arterial and portal blood flow by xenon computed tomography: Quantitative analysis of correlation with hepatic fibrosis in chronic hepatitis C. Hepatology. 38 Supplement: 421

[26] Jaeschke H, Gores GJ, Cederbaum AI, Hinson JA, Pessayre D, et al (2002) Mechanisms of hepatotoxicity. Toxicol Sci 65: 166–176. 11812920 10.1093/toxsci/65.2.166

[27] Manibusan MK, Odin M, Eastmond DA (2007) Postulated carbon tetrachloride mode of action: a review. J Environ Sci Health C Environ Carcinog Ecotoxicol Rev 25: 185–209. 17763046 10.1080/10590500701569398

[28] Chan WH, Liao JW, Chou CP, Chan PK, Wei CF, et al (2009) Induction of CYP1A1, 2B, 2E1 and 3A in rat liver by organochlorine pesticide dicofol. Toxicol Lett 190: 150–155. 19595748 10.1016/j.toxlet.2009.07.005

[29] Dutta SK, Ghosh S, De S, Hoffman EP (2008) CYP1A1 and MT1K are congener specific biomarker genes for liver diseases induced by PCBs. Environ Toxicol Pharmacol 25: 218–221. 21783860 10.1016/j.etap.2007.10.018

[30] Moon YJ, Wang X, Morris ME (2006) Dietary flavonoids: effects on xenobiotic and carcinogen metabolism. Toxicol In Vitro 20: 187–210. 16289744 10.1016/j.tiv.2005.06.048

[31] Boll M, Weber LW, Becker E, Stampfl A (2001) Hepatocyte damage induced by carbon tetrachloride: inhibited lipoprotein secretion and changed lipoprotein composition. Z Naturforsch C 56: 283–290. 11371022 10.1515/znc-2001-3-419

[32] Fang Y-Z, Yang S, Wu G (2002) Free radicals, antioxidants, and nutrition. Nutrition 18: 872–879. 12361782 10.1016/s0899-9007(02)00916-4

[33] Ghiselli A, Serafini M, Natella F, Scaccini C (2000) Total antioxidant capacity as a tool to assess redox status: critical view and experimental data. Free Radic Biol Med 29: 1106–1114. 11121717 10.1016/s0891-5849(00)00394-4

[34] Bandy B, Bechara EJ (2001) Bioflavonoid rescue of ascorbate at a membrane interface. J Bioenerg Biomembr 33: 269–277. 11710803 10.1023/a:1010641422120

[35] Racanelli V, Rehermann B (2006) The liver as an immunological organ. Hepatology 43: S54–62. 16447271 10.1002/hep.21060

[36] Leung TM, Fung ML, Liong EC, Lau TY, Nanji AA, et al (2010) Role of nitric oxide in the regulation of fibrogenic factors in experimental liver fibrosis in mice. Histol Histopathol 26: 201–211. 10.14670/HH-26.201 21154234

[37] Zimiani K, Guarnier FA, Miranda HC, Ehara Watanabe MA, Cecchini R (2005) Nitric oxide mediated oxidative stress injury in rat skeletal muscle subjected to ischemia/reperfusion as evaluated by chemiluminescence. Nitric Oxide 13: 196–203. 16125423 10.1016/j.niox.2005.07.002

[38] Zhu W, Fung PC (2000) The roles played by crucial free radicals like lipid free radicals, nitric oxide, and enzymes NOS and NADPH in CCl(4)-induced acute liver injury of mice. Free Radic Biol Med 29: 870–880. 11063912 10.1016/s0891-5849(00)00396-8

[39] Hu K-Q (2003) Cyclooxygenase 2 (COX2)-prostanoid pathway and liver diseases. Prostaglandins Leukot Essent Fatty Acids 69: 329–337. 14580367 10.1016/j.plefa.2003.07.001

[40] Sunami Y, Leitháuser F, Gul S, Fiedler K, Güldiken N, et al (2012) Hepatic activation of Ikk/NF-kB signaling induces liver fibrosis via macrophage-mediated chronic inflammation. J Hepatol 56 Supplement 2S160. 10.1002/hep.25711 22407857

[41] Palanisamy N, Kannappan S, Anuradha CV (2011) Genistein modulates NF-κB-associated renal inflammation, fibrosis and podocyte abnormalities in fructose-fed rats. Eur J Pharmacol 667: 355–364. 21704028 10.1016/j.ejphar.2011.06.011

[42] Muriel P (2009) NF-kappaB in liver diseases: a target for drug therapy. J Appl Toxicol 29: 91–100. 18937212 10.1002/jat.1393

[43] Castello L, Froio T, Maina M, Cavallini G, Biasi F, et al (2010) Alternate-day fasting protects the rat heart against age-induced inflammation and fibrosis by inhibiting oxidative damage and NF-kB activation. Free Radic Biol Med 48: 47–54. 19818847 10.1016/j.freeradbiomed.2009.10.003

[44] Gloire G, Legrand-Poels S, Piette J (2006) NF-κB activation by reactive oxygen species: fifteen years laterBiochem Pharmacol. 72: 1493–1505. 10.1016/j.bcp.2006.04.011 16723122

[45] Morgan MJ, Liu Z-g (2010) Crosstalk of reactive oxygen species and NF-κB signaling. Cell Res 21: 103–115. 21187859 10.1038/cr.2010.178 PMC3193400

[46] Liu Y, Wang Z, Kwong SQ, Lui ELH, Friedman SL, et al (2011) Inhibition of PDGF, TGF-β, and Abl signaling and reduction of liver fibrosis by the small molecule Bcr-Abl tyrosine kinase antagonist Nilotinib. J Hepatol 55: 612–625. 21251937 10.1016/j.jhep.2010.11.035

[47] Okamoto K, Mimura K, Murawaki Y, Yuasa I (2005) Association of functional gene polymorphisms of matrix metalloproteinase (MMP)-1, MMP-3 and MMP-9 with the progression of chronic liver disease. J Gastroenterol Hepatol 20: 1102–1108. 15955221 10.1111/j.1440-1746.2005.03860.x

[48] Veidal SS, Karsdal MA, Nawrocki A, Larsen MR, Dai Y, et al (2011) Assessment of proteolytic degradation of the basement membrane: a fragment of type IV collagen as a biochemical marker for liver fibrosis. Fibrogenesis Tissue Repair 4: 22. 21970406 10.1186/1755-1536-4-22 PMC3204229

[49] Theret N, Lehti K, Musso O, Clement B (1999) MMP2 activation by collagen I and concanavalin A in cultured human hepatic stellate cells. Hepatology 30: 462–468. 10421655 10.1002/hep.510300236

[50] Belotti D, Paganoni P, Manenti L, Garofalo A, Marchini S, et al (2003) Matrix Metalloproteinases (MMP9 and MMP2) Induce the Release of Vascular Endothelial Growth Factor (VEGF) by Ovarian Carcinoma Cells. Cancer Res 63: 5224–5229. 14500349

[51] Vassiliadis E, Larsen DV, Clausen RE, Veidal SS, Barascuk N, et al (2011) Measurement of CO3–610, a potential liver biomarker derived from matrix metalloproteinase-9 degradation of collagen type iii, in a rat model of reversible carbon-tetrachloride-induced fibrosis. Biomark Insights 6: 49–58. 21499440 10.4137/BMI.S6347 PMC3076019

[52] Han YP, Tuan TL, Hughes M, Wu H, Garner WL (2001) Transforming growth factor-beta - and tumor necrosis factor-alpha -mediated induction and proteolytic activation of MMP-9 in human skin. J Biol Chem 276: 22341–22350. 11297541 10.1074/jbc.M010839200 PMC2651823

